# Chronic Light-Induced Desynchronosis as a Model of Accelerated Metabolic Aging in Rats: Prevention and Correction by Exogenous Melatonin

**DOI:** 10.3390/biomedicines14061303

**Published:** 2026-06-08

**Authors:** David A. Areshidze, Maria A. Kozlova, Anna I. Anurkina, Valery P. Chernirov

**Affiliations:** Avtsyn Research Institute of Human Morphology, B.V. Petrovsky Russian Scientific Center of Surgery, Moscow 119991, Russia; kma-morph@mail.ru (M.A.K.);

**Keywords:** circadian disruption, hepatoprotection, melatonin, chronobiotic agents, metabolic dysfunction, light pollution

## Abstract

**Background:** Chronic exposure to artificial light at night (light pollution) causes circadian desynchronosis and melatonin deficiency, which is considered an independent driver of metabolic disorders and accelerated aging. However, the long-term effects of chronic desynchronosis on systemic metabolism and liver structure throughout the life cycle, as well as the potential of preventive melatonin administration, remain poorly understood. **Objective:** To evaluate the effects of chronic dark deprivation and prevention of metabolic disorders by exogenous melatonin on plasma melatonin levels, metabolic profile, liver function, and morphological changes in rats over a 24-month experiment. **Methods:** A 24-month experiment was conducted on 360 male Wistar rats divided into three groups: control (standard 10:14 h light/dark photoperiod), dark deprivation (DD, constant illumination), and correction (DD+Mel, constant illumination + melatonin 10 mg/kg five times per week). Animals were sacrificed at 6, 12, 18, and 24 months. Plasma melatonin was assessed by ELISA. Biochemical parameters (ALT, AST, LDH, total protein, albumin, bilirubin, glucose, triglycerides, and cholesterol), body weight, liver weight, relative liver weight, and histological parameters (steatosis, fibrosis, nuclear area, nuclear/cytoplasmic ratio, and binucleated hepatocytes) were analyzed. **Results:** In the DD group, a persistent progressive melatonin deficiency was detected (5.1-fold decrease by 6 months, *p* < 0.0005), accompanied by hypertriglyceridemia (Cohen’s d = 6.40), hypercholesterolemia (d = 4.59), biphasic dysglycemia (hypoglycemia followed by hyperglycemia), elevated ALT and AST activity (d = 2.60 and 2.46, respectively), hypoproteinemia (d = 5.33), hypoalbuminemia (d = 3.34), and hyperbilirubinemia (d = 3.22–4.37), as well as progressive steatosis (2.8 ± 0.3 points, d = 7.20) and pericellular fibrosis (1.8 ± 0.4 points, d = 4.50). In the DD group, a decrease in relative liver weight during the first 12 months was observed (metabolic disproportion, d = 2.31), reflecting disproportionate body weight gain. In the DD+Mel group, exogenous melatonin restored the biochemical parameters to values that did not differ statistically from the control values (Cohen’s d < 0.2 for most parameters), prevented steatosis (0.8 ± 0.3 points, d = 0.80) and fibrosis (0 points), increased relative liver weight by 24 months (3.83 ± 0.49 vs. 3.27 ± 0.029 in the control, d = 1.60), and increased the hepatocyte nuclear area (58.4 ± 4.1 vs. 48.6 ± 3.8 μm^2^, d = 2.32). **Conclusions:** Chronic desynchronosis induced by constant illumination leads to persistent melatonin deficiency and complex metabolic and structural liver disturbances modeling accelerated aging. Exogenous melatonin (10 mg/kg five times per week) exhibits pronounced geroprotective, hepatoprotective, and antifibrotic effects, normalizing all biochemical parameters and preventing age-related liver involution.

## 1. Introduction

Aging is a complex biological process characterized by the gradual accumulation of damage at the molecular, cellular, and tissue levels, which ultimately leads to a systemic decline in physiological functions and the development of age-associated diseases [1]. In the modern world, along with genetic and metabolic factors, the role of chronobiological disturbances as an independent driver of accelerated aging is attracting increasing attention [2,3]. One of the most common socially significant disturbances of this kind is so-called “light pollution”—chronic exposure to artificial light at night caused by urbanization, the use of electronic devices, and shift work [4,5].

The pathogenic effect of light pollution is primarily mediated through the disintegration of the body’s circadian system. The central element of this system is the pineal gland, which secretes melatonin, a key hormone regulating daily rhythms—a “darkness signal” for the entire body [6,7]. Melatonin synthesis and secretion are strictly inhibited by light, which leads to a persistent and pronounced deficiency of this hormone under conditions of round-the-clock illumination [8]. In addition to its chronobiotic function, melatonin possesses powerful pleiotropic properties, acting as a universal antioxidant [9,10,11], anti-inflammatory agent [12,13], and immunomodulator [14,15].

The liver, being a central metabolic organ whose activity is under strict circadian control, is particularly vulnerable to the consequences of desynchronosis [16,17]. The expression of up to 80% of genes in hepatocytes, including those responsible for lipid and carbohydrate metabolism and detoxification, exhibits pronounced daily rhythmicity [18,19]. Disruption of circadian regulation in the liver is a key element in the pathogenesis of non-alcoholic fatty liver disease (NAFLD), insulin resistance, and fibrosis [20,21]. It has been shown that knockout of key clock genes, such as *Bmal1* and *Clock*, leads to pronounced metabolic disturbances and steatosis [22].

Despite accumulated evidence on the link between desynchronosis, melatonin deficiency, and liver pathology, several fundamental questions remain poorly understood. Most experimental studies are short-term and do not cover the dynamics of changes throughout the entire life cycle of the organism. Furthermore, existing studies often focus either on the molecular mechanisms of clock gene function or on individual metabolic parameters without an integrated assessment of the complex biochemical status and morphological changes occurring in the liver. The most important question is to what extent correction of melatonin deficiency can mitigate desynchronosis-induced systemic metabolic and organ disorders, including structural changes in the liver.

The selected dose of melatonin (10 mg/kg, five times per week) is supraphysiological and corresponds to pharmacological replacement rather than mere restoration of endogenous levels, which is necessary to achieve a geroprotective effect under conditions of complete pineal exhaustion [23].

The aim of this study was to conduct a comprehensive longitudinal assessment of the effects of chronic dark deprivation and subsequent correction with exogenous melatonin on the dynamics of plasma melatonin levels, markers of liver functional status, general metabolic profile, and anthropometric and histological parameters in rats over a 24-month experiment.

## 2. Materials and Methods

### 2.1. Study Design

A 24-month experiment was conducted on 360 male Wistar rats (initial body weight 200–220 g). The animals were obtained from the Stolbovaya nursery of the Federal State Budgetary Institution of Science “Scientific Center of Biomedical Technologies” of the Federal Medical and Biological Agency of Russia. All animal procedures were performed in accordance with the European Convention for the Protection of Vertebrate Animals used for Experimental and Other Scientific Purposes (Strasbourg, 1986) and were approved by the Bioethics Committee of the Avtsyn Research Institute of Human Morphology (Protocol No. 34(10) dated 14 March 2021).

After a one-week adaptation period in the vivarium, the rats were randomly assigned to three groups (n = 120 each):

Control group (Control)—with standard vivarium conditions and a fixed photoperiod of light/darkness 10:14 h (lights on at 08:00, lights off at 18:00).

Dark deprivation group (DD)—with constant round-the-clock illumination to induce chronic desynchronosis and suppress endogenous melatonin secretion.

Correction group (DD+Mel)—with constant illumination + exogenous melatonin (Sigma-Aldrich, St. Louis, MO, USA, catalog number M5250) at a dose of 10 mg/kg body weight 5 times per week [18].

No separate vehicle control group (constant illumination + water without melatonin) was included because the DD group itself received clean water under constant illumination. However, we acknowledge that the taste and smell of melatonin solution could affect fluid intake, although no significant differences in water consumption were observed in our previous studies.

The primary endpoint was the presence and severity of hepatic steatosis and fibrosis. Secondary endpoints included plasma melatonin levels, biochemical markers of liver function (ALT, AST, LDH, total protein, albumin, and bilirubin), metabolic parameters (glucose, triglycerides, and cholesterol), body weight, liver weight, relative liver weight, and morphometric parameters (hepatocyte nuclear area, nuclear/cytoplasmic ratio, and proportion of binucleated hepatocytes).

No formal a priori sample size calculation was performed. The sample size (n = 120 per group initially, with n = 16–22 animals per group at each time point) was chosen based on previously published longitudinal studies in rat models of aging and circadian disruption [24,25], which demonstrated that such numbers are sufficient to detect large effect sizes (Cohen’s d > 0.8) for primary outcomes (melatonin level, liver steatosis, and fibrosis) with a power > 0.8 at α = 0.05. The final number of animals at each time point accounted for anticipated natural mortality (approximately 15–20% by 24 months), which was consistent with the observed survival.

All animals that survived to the scheduled euthanasia time points were included in the analysis. Exclusion criteria were defined a priori as: (a) death before the scheduled euthanasia (natural mortality); (b) visible tumors or severe organ pathology (e.g., polycystic liver, large cysts, abscesses) detected at necropsy; and (c) signs of fighting or cannibalism (wounds, missing body parts). No animals were excluded for behavioral reasons or outliers in biochemical parameters. In the DD+Mel group, animals that consistently did not drink the melatonin solution (based on a daily water intake < 5 mL per 100 g body weight) were planned to be excluded, but none met this criterion.

Illumination was provided by halogen lamps with a light output of 9–19 lm/W, a color temperature of 2860–4400 K, an emission spectrum of 460–660 nm, and a biological equivalent (BioEq) of 100%. Illuminance at the eye level of the laboratory animals was measured using a TKA-LUX lux meter (NTP TKA, Moscow, Russia). For animals in the control and correction groups, illuminance at the cage level was 760 lx/m^2^. During the night hours, illuminance in the control group was 0–1 lx/m^2^. All animals were kept in standard plastic cages (5 animals per cage) at a controlled temperature (22 ± 2 °C) and air humidity (60 ± 10%). The rats had free access to pelleted compound feed for laboratory animals PK-120-1 (Laboratorsnab LLC, Moscow, Russia) and drinking water (in the control and DD groups, clean water, and in the DD+Mel group, an aqueous solution of melatonin).

For dynamic assessment of the studied parameters, animals were sacrificed at four time points: 6 months (young adult age), 12 months (adult age), 18 months (elderly age), and 24 months (old age). Animals were removed from the experiment using the time series method (regular time intervals) four times a day at 9:00, 15:00, 21:00, and 3:00, with 10–8 animals sacrificed at each time point to ensure the statistical validity of the results. After sacrifice, a necropsy was performed. The number of animals in the groups at the time of sampling decreased due to natural mortality. In the control group, 22 rats were examined at 18 months, and 18 rats at 24 months; in the DD group, 19 and 16 animals, respectively; in the DD+Mel group, 20 animals at 24 months.

### 2.2. Anthropometric Parameters

Twelve hours before sampling, animals were deprived of food to standardize their metabolic status, while free access to water was maintained. Immediately before euthanasia, animals were weighed on electronic scales with an accuracy of 0.1 g (Mettler Toledo, Greifensee, Switzerland). Euthanasia was performed by a gradual filling of the chamber with carbon dioxide (CO_2_) at a rate of 20% of the chamber volume per minute.

Immediately after confirmation of death, a complete necropsy was performed. The liver was removed and weighed on an analytical balance with an accuracy of 0.01 g (Sartorius, Göttingen, Germany). Relative liver weight (index) was calculated using the following formula: Relative liver weight = (liver weight, g/body weight, g) × 100% [26].

### 2.3. Biochemical Methods

Blood collection was performed from the inferior vena cava into vacuum tubes containing K_2_EDTA (Vacuette, Greiner Bio-One, Kremsmünster, Austria). Blood samples were centrifuged at 3000 rpm for 15 min at +4 °C to obtain plasma. Plasma was immediately aliquoted and frozen at −80 °C.

The plasma melatonin concentration was determined using the commercial solid-phase enzyme-linked immunosorbent assay kit, “ELISA Kit for Melatonin (MLT)” (Cat. No. CEA908Ge, Cloud-Clone Corp., Wuhan, China), according to the manufacturer’s instructions.

The biochemical parameters of liver function (alanine aminotransferase—ALT; aspartate aminotransferase—AST; lactate dehydrogenase—LDH; alkaline phosphatase—ALP; and total protein, albumin, total, and direct bilirubin) and metabolic status (glucose, triglycerides, total cholesterol) were measured using an automatic biochemical analyzer, the “Cormay Accent 200” (Cormay, Lublin, Poland), with standard reagents from the manufacturer.

### 2.4. Histological Examination of the Liver

After weighing, fragments measuring 5 × 5 × 3 mm were excised from the cranial portion of the right lateral lobe of the liver for histological examination. The fragments were fixed in 10% neutral buffered formalin (pH 7.4) for 24 h, then processed through a series of alcohols of increasing concentration (70°, 80°, 96°, and 100°), and embedded in paraffin (Histomix, BioVitrum, Saint Petersburg, Russia). Sections of 5 μm thickness were prepared using a rotary microtome (Leica RM2235, Nussloch, Germany).

For general analysis, sections were stained with hematoxylin and eosin. To detect steatosis, staining with Sudan III black on cryostat sections (10 μm thickness) was used.

### 2.5. Morphometric Analysis

From each animal, we took at least three sections from different liver lobes (the left, right, and caudate lobes). From each liver block (5 × 5 × 3 mm), a series of non-overlapping sections, 5 µm thick, was cut at intervals of at least 200 µm to avoid double-counting the same microscopic structures. For each animal, 10 sections were prepared, and in each section, 5 fields of view (×200 magnification) were analyzed. Thus, a total of 50 fields of view per animal were evaluated for morphometric parameters. For nuclear area measurement and binucleated hepatocyte counting, at least 250 hepatocytes per animal were measured. The following parameters were evaluated: the area of hepatocyte nuclei and the area of the hepatocytes themselves (μm^2^), with at least 250 nuclei and cells per animal measured; the nuclea/cytoplasmic ratio (NCR), the ratio of nuclear area to cytoplasmic area; and the proportion of binucleated hepatocytes (% of total hepatocytes), a marker of regenerative activity.

The degree of steatosis and the severity of fibrosis were assessed using a scoring system (0–3 points):

0 points—absence;

1 point—mild (less than 30% of hepatocytes affected);

2 points—moderate (30–60% of hepatocytes affected);

3 points—severe (more than 60% of hepatocytes affected).

The experiment was not fully blinded due to the obvious difference in lighting conditions (constant light vs. 10:14 h photoperiod) and the presence of melatonin in drinking water (which could be detected by the bitter taste). However, outcome assessments were performed blinded to group allocation as follows: (a) blood samples were coded and analyzed for melatonin and biochemical parameters by a technician who had no knowledge of the experimental groups; (b) histological slides were randomized and evaluated by two independent pathologists who were blinded to the group identity (the code was broken only after all morphometric measurements were completed). Inter-observer agreement for steatosis and fibrosis scores was high (Cohen’s κ = 0.87 for steatosis, and 0.92 for fibrosis).

### 2.6. Statistical Analysis

Statistical data processing was performed using GraphPad Prism v8.41 software (GraphPad Software, Boston, MA, USA) and IBM SPSS Statistics v26.0 (IBM Corp., Armonk, NY, USA). Data are presented as an arithmetic mean and standard deviation (M ± SD) for parametric data, and as a median and interquartile range (Me [Q1; Q3]) for non-normally distributed data.

The normality of distribution of quantitative traits was tested using the Shapiro–Wilk test (for n < 50) and the Kolmogorov–Smirnov test with Lilliefors correction (for n ≥ 50). The distribution was considered normal at *p* > 0.05.

For a comparison of parameters between the three groups at each age point, the following were used:

Since independent groups of animals were used for each time point (cross-sectional design), the analysis was carried out using two-way ANOVA with independent factors “time” (4, 8, 12, 24 months, etc.) and “treatment” (control, constant light, constant light + melatonin). In the absence of a significant interaction between factors, the main effects were evaluated. When a significant interaction was present, post hoc comparisons were performed using Tukey’s HSD test for multiple comparisons between groups at each individual time point. For comparisons of only two independent groups at a single time point, an unpaired *t*-test (for normally distributed data) or the Mann–Whitney U test (for non-normally distributed data) was used. The significance threshold was set at *p* < 0.05.

The non-parametric Kruskal–Wallis test with Dunn’s post hoc test for multiple comparisons was used when normality of distribution or homogeneity of variances was violated.

To assess the dynamics of changes within each group throughout ontogenesis (4 time points: 6, 12, 18, and 24 months), the following were used:

Friedman’s test with Dunn’s post hoc test for non-normally distributed data.

To control for type I errors in multiple comparisons, the Holm–Bonferroni correction was applied. Differences were considered statistically significant at *p* < 0.05 after correction.

To assess the clinical and biological significance of the identified differences, in addition to the level of statistical significance (*p*-value), the effect size according to Cohen (Cohen’s d) was calculated. This parameter allows for a quantitative assessment of the magnitude of differences between groups, independent of the sample size.

Cohen’s d was calculated using the following formula:

d = (M_1_ − M_2_)/SD_pooled

where

M_1_, M_2_ are the mean values of the compared groups;

SD_pooled is the pooled standard deviation: √[(SD_1_^2^ + SD_2_^2^)/2].

The calculation was performed for the following pairwise comparisons:

DD vs. Control to assess the magnitude of metabolic and morphological disturbances induced by chronic desynchronosis;

DD+Mel vs. Control to assess the degree of normalization of parameters during exogenous melatonin therapy.

Cohen’s d was considered positive (the direction of the effect was indicated) if the differences corresponded to a worsening of the parameter in the DD group compared to the control, or if melatonin therapy led to an improvement of the parameter relative to the control.

## 3. Results

### 3.1. Dynamics of Plasma Melatonin Levels

**Figure 1:**
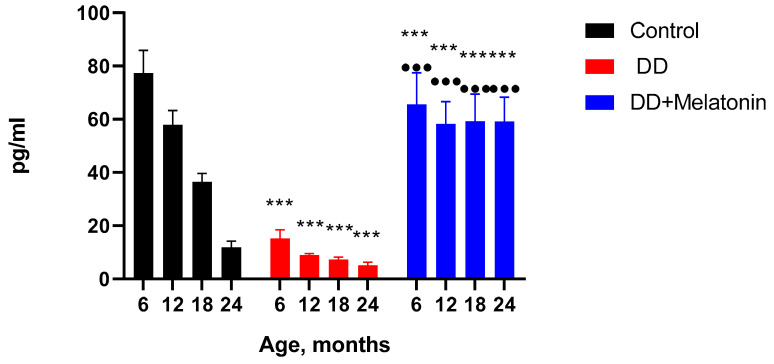
Dynamics of melatonin concentration in the blood of rats during ontogenesis. Note. *** (*p* ≤ 0.0005): in comparison with control values during the same period of ontogenesis; ●●● (*p* ≤ 0.0005): in comparison with DD group values during the same period of ontogenesis.

The results of the enzyme immunoassay revealed a pronounced effect of dark deprivation and exogenous melatonin on plasma hormone levels (Figure 1).

In the control group, a physiological age-related dynamic was observed: the maximum melatonin concentration was recorded at 6 months of age (77.29 ± 8.59 pg/mL), followed by a progressive, significant decrease by 12 months (57.85 ± 5.44 pg/mL), 18 months (30.44 ± 3.21 pg/mL), and 24 months (11.91 ± 2.31 pg/mL). By the end of the experiment, the hormone level had decreased 6.5-fold compared to the initial level (Figure 1, Table 1).

In the dark deprivation (DD) group, a radically different pattern was observed. Already at 6 months, the melatonin concentration was extremely low (15.18 ± 3.27 pg/mL) and significantly inferior to the control values. In subsequent age periods, the hormone level continued to decrease statistically significantly, reaching a minimum value of 5.15 ± 1.11 pg/mL by 24 months. Thus, constant illumination not only caused acute suppression of melatonin synthesis at a young age but also prevented its physiological increase, leading to a persistent and progressive deficiency.

In the DD+Melatonin group, the administration of exogenous melatonin ensured the maintenance of a consistently high hormone concentration at all stages of ontogenesis. Although at 6 months the level was somewhat lower than in the control (68.4 ± 5.2 pg/mL), starting from 12 months until the end of the experiment, the melatonin level in this group did not differ significantly from that of young control animals and significantly exceeded the levels of both the aging control and the DD groups.

### 3.2. Dynamics of Body Weight, Liver Weight, and Relative Liver Weight

In the control group, a classic age-related dynamic was observed: maximum body weight was recorded at 18 months, followed by a significant decrease by 24 months, reflecting age-related sarcopenia and a general decline in metabolic activity in old age. In the dark deprivation (DD) group, body weight was significantly higher than the control values at all age points, reaching a maximum by 24 months, whereas in the control group, body weight had already decreased by this time. Animals in the correction group (DD+Mel) occupied an intermediate position: their body weight was significantly lower than in the DD group at all ages except 24 months, but was higher than the control at 6 and 12 months, and decreased to the control level by 24 months (Table 2).

In the control group, liver weight naturally increased until 18 months, after which it decreased by 24 months. In the DD group, liver weight was consistently higher than in the control at all age points. In the DD+Mel group, liver weight did not differ from the control at 6 and 12 months, but at 18 months it was significantly higher than both the control and the DD groups, after which it decreased by 24 months.

Relative liver weight (index), reflecting the proportionality of organ and body development, demonstrated the most informative dynamics. In the control group, the index remained stable during the period of active growth, then sharply decreased by 24 months, reflecting age-related liver atrophy and a decline in its metabolic activity in old age.

In the DD group, a sustained decrease in relative liver weight was observed during the first 12 months. This indicates that under the influence of dark deprivation, body weight grew at a faster rate compared to liver weight. Only by 18 and 24 months did the index in the DD group reach control values; however, by 24 months, it had decreased again and did not differ from the control. In the DD+Mel group, the pattern was fundamentally different. After some decrease in the index by 12 months, by 18 months the value recovered (3.82 ± 0.97), and by 24 months it significantly increased compared to the control and the DD groups, reaching the maximum value among all groups.

### 3.3. Biochemical Parameters

*Cytolysis enzymes (ALT, AST, LDH)*. In the control group, ALT and AST activity naturally increased with age, reflecting the accumulation of age-related damage to hepatocytes. In the DD group, ALT activity was significantly higher than in the control at all age points, reaching a maximum by 24 months. A similar dynamic was observed for AST. LDH activity in the DD group was also higher than in the control at all time points, showing a maximum increase at 12 months. In the DD+Mel group, the activity of all three enzymes was significantly lower than in the DD group and practically did not differ from the control values. This indicates effective protection of hepatocyte membranes by exogenous melatonin.

*Protein-synthetic function.* In the control group, total protein and albumin levels remained stable until 18 months, followed by a decrease by 24 months. In the DD group, persistent and progressive hypoproteinemia and hypoalbuminemia were revealed. In the DD+Mel group, total protein and albumin levels at all stages did not significantly differ from the control values but were significantly higher than those in the DD group (Table 2).

*Bilirubin (cholestasis).* In the control group, a moderate age-related increase in total and direct bilirubin was observed. In the DD group, these changes were significantly more pronounced: total bilirubin reached 9.16 ± 0.88 μmol/L by 24 months. In the DD+Mel group, bilirubin levels did not differ from the control values throughout most of the experiment.

*Carbohydrate metabolism.* In the control group, a gradual age-related increase in glucose levels was observed. In the DD group, a characteristic biphasic pattern was revealed: hypoglycemia at a young age, which later turned into pronounced and progressive hyperglycemia by 12 and 24 months. In the DD+Mel group, melatonin completely restored the glycemic profile: parameters at all stages, except for 6 months, did not statistically differ from the control values and were significantly lower than in the DD group.

*Lipid metabolism.* In the control group, cholesterol levels increased from 58.59 ± 4.83 mg/dL at 6 months to 87.0 ± 13.83 mg/dL at 24 months. In the DD group, progressive hypertriglyceridemia and hypercholesterolemia were revealed. In the DD+Mel group, both parameters practically did not differ from the control values throughout ontogenesis (Table 3).

### 3.4. Histological Examination of the Liver

The results of the histological and morphometric examination of the liver are presented in Table 4.

At the ages of 6 and 12 months, the histoarchitecture of the liver in the control group was preserved: hepatic beams radially converged to the central vein, hepatocytes had a polygonal shape with clear boundaries, and the cytoplasm was eosinophilic. Signs of steatosis and fibrosis were absent. By 18 months, single hepatocytes with signs of hydropic degeneration appeared; the degree of steatosis was 0.5 ± 0.2 points. By 24 months, moderate atrophy of the hepatic beams and dilation of the sinusoids were noted; steatosis reached 1.0 ± 0.2 points, and single lymphohistiocytic infiltrates appeared in the portal tracts. Fibrosis was absent throughout all age periods.

In animals of the DD group, already at 6 months, signs of dysregeneration were observed, such as an increase in the number of binucleated hepatocytes and focal vacuolization of the cytoplasm. The degree of steatosis at 6 months was 1.0 ± 0.3 points. By 12 months, steatosis progressed to 2.5 ± 0.4 points, and focal lymphohistiocytic infiltrates appeared. By 18 months, pronounced balloon degeneration of hepatocytes, dilation of the Disse spaces, and moderate pericellular fibrosis were noted. By 24 months, steatosis reached 2.8 ± 0.3 points, fibrosis reached 1.8 ± 0.4 points, and porto-portal septa were observed.

In the DD+Mel group, at 6 and 12 months, the histological picture practically did not differ from the control: steatosis was absent (0–0.2 points), there was no fibrosis, and the area of hepatocyte nuclei was normal. By 18 months, a slight increase in the number of binucleated hepatocytes was noted, but signs of dystrophy and necrosis were absent. By 24 months, steatosis was 0.8 ± 0.3 points, and fibrosis was absent (0 points). The most important observation in the DD+Mel group by 24 months was an increase in the area of hepatocyte nuclei (58.4 ± 4.1 μm^2^) compared to the control and the DD groups, indicating nuclear hypertrophy and, probably, increased transcriptional activity.

## 4. Discussion

In this 24-month longitudinal study, we provide a comprehensive assessment of chronic light-induced desynchronosis on rat liver metabolism, function, and morphology, and demonstrate the high effectiveness of exogenous melatonin (10 mg/kg, five times/week). The integration of biochemical, anthropometric, histological, and statistical methods (including Cohen’s d) revealed a holistic picture of structural and functional liver changes across the life cycle.

Chronic constant illumination caused a persistent, progressive decrease in plasma melatonin (see Results). The dose of melatonin used (10 mg/kg, five times/week) is supraphysiological. The oral bioavailability of melatonin in rats is approximately 53.5%, and the half-life is about 20 min [27]. Thus, a high dose is required to maintain therapeutic levels throughout the dark phase. The observed “better-than-control” effects (increased relative liver weight and nuclear area) suggest pharmacological enhancement rather than mere restoration. In the DD group, we identified a novel phenomenon of metabolic disproportion: a disproportionate increase in body weight relative to liver weight during the first 12 months (very large effect size, d = 2.31). This suggests that the liver fails to adapt to increased metabolic demands under desynchronosis. Constant light also induced hypertriglyceridemia, hypercholesterolemia, biphasic dysglycemia, elevated transaminases and LDH, hypoproteinemia, hypoalbuminemia, hyperbilirubinemia, progressive steatosis, and pericellular fibrosis with porto-portal septa (for the extreme effect sizes, see Section 3). Morphometric analysis revealed a reduced hepatocyte nuclear area and increased binucleated cells, reflecting persistent regenerative stress.

In the DD+Mel group, exogenous melatonin prevented hormone deficiency, restored all biochemical parameters (Cohen’s d mostly < 0.2), and prevented steatosis (d for improvement 0.80) and fibrosis (complete absence). Moreover, by 24 months, relative liver weight and hepatocyte nuclear area were increased compared to the controls (very large positive effects). These data indicate that chronic desynchronosis is an independent driver of accelerated metabolic aging, and melatonin replacement exerts pronounced geroprotective, hepatoprotective, and antifibrotic actions.

Our results confirm that suppression of melatonin secretion is key in chronic light pollution. Constant illumination (760 lx/m^2^) inhibits AANAT by 80–95% [8,28], which is consistent with our very low melatonin levels in DD (see the Results section). The physiological age-related decline in control rats (6.5-fold over 24 months) matches the concept of the pineal as an aging clock [29,30,31]. Under desynchronosis, this decline becomes catastrophic, depleting antioxidant and metabolic reserves (extreme effect sizes for triglycerides, steatosis, and fibrosis). In DD+Mel, consistently high melatonin (68–115 pg/mL) was maintained, exceeding the aging control levels and creating a “therapeutic excess”.

Metabolic disproportion (a decreased relative liver weight in DD during the first 12 months) is a novel observation. In the controls, relative liver weight remained stable, indicating harmonious growth. In DD, extreme body weight gain (d = 4.30 at 6 months) contrasted with slow liver growth, meaning that melatonin deficiency and circadian stress favor fat/muscle gain while the liver’s synthetic detoxification apparatus lags. This agrees with the literature: desynchronosis accelerates visceral obesity [32], and shift work increases the risk of metabolic syndrome [33,34]. Mechanisms include disrupted leptin/ghrelin regulation [35], reduced UCP1-mediated thermogenesis [26,36,37], and dysregulated SREBP-1c/PPARγ [21,38]. In DD+Mel, metabolic disproportion was prevented, and relative liver weight at 24 months exceeded the control values, which is strong evidence of geroprotection.

The DD group for dyslipidemia (extreme effect sizes for triglycerides and cholesterol) indicates a profound impact on lipid metabolism due to a high-fat diet or genetic models. Mechanisms include the following: (a) a dysregulated SREBP-1c with constitutive activation of FAS and ACC [21,38,39]; (b) impaired β-oxidation due to reduced AMPK/PPARα [40,41,42]; (c) increased lipolysis from disrupted leptin/ghrelin [43,44]; and (d) decreased lipoprotein clearance [43,45,46]. In DD+Mel, lipid parameters were restored to levels not differing statistically from the controls (d = 0.09–0.25 for cholesterol; d = 0.00–0.19 for triglycerides at 6–18 months). At 24 months, triglycerides were even lower than the control (d = 2.42 toward improvement), suggesting an additional lipid-lowering action that is independent of desynchronosis correction [46,47].

Glycemic dynamics in DD showed a biphasic pattern: hypoglycemia at a young age followed by hyperglycemia (very large effect sizes). This reflects stepwise carbohydrate dysregulation [48,49,50]: Phase 1 (compensatory hypoglycemia), possibly due to increased insulin secretion or sensitivity; Phase 2 (decompensation) as β-cells exhaust and insulin resistance develops. Melatonin normalizes insulin secretion rhythms and improves insulin sensitivity [48,49,51]. Clinical data link low melatonin to type 2 diabetes risk [31]. In DD+Mel, glycemia was fully restored (d = 0.08–0.19).

ALT, AST, and LDH activities in DD were markedly elevated (very large to extreme effect sizes), indicating progressive hepatocyte membrane damage from oxidative stress, inflammation, and apoptosis [44,52]. In DD+Mel, enzyme activities were near the control values (d = 0.07–0.68), consistent with clinical meta-analyses showing melatonin reduces ALT/AST in NAFLD [53,54,55].

The DD group exhibited persistent hypoproteinemia and hypoalbuminemia (extreme effect sizes), reflecting suppressed protein synthesis due to ribosome loss, ATP deficiency, and acute-phase protein switching [25,52]. In DD+Mel, protein and albumin levels did not differ from the controls (d = 0.04–0.48), demonstrating preservation of synthetic function.

The DD group also showed marked hyperbilirubinemia (very large to extreme effect sizes), suggesting a cholestatic component from oxidative damage to transport proteins (BSEP, MRP2, MDR3) [56]. In DD+Mel, bilirubin remained normal (d = 0.20–0.60), likely via antioxidant protection or Nrf2 activation [57].

Liver steatosis in DD progressed to extreme severity (d = 3.90–7.67), proving that chronic melatonin deficiency independently drives NAFLD without dietary triggers. Mechanisms involve increased de novo lipogenesis (SREBP-1c) [21,38], reduced β-oxidation [50,51], and increased free fatty acid flux [43]. In DD+Mel, steatosis was minimal (d = 0.80 improvement), which is consistent with previous studies [13,58,59].

Fibrosis was the most dramatic change: in DD, pericellular fibrosis reached 1.8 points (d = 4.50, extreme effect) with porto-portal septa (METAVIR F2-F3). This is the first experimental evidence that light-induced desynchronosis alone can induce liver fibrosis. Mechanisms include HSC activation by oxidative stress [60], chronic inflammation with cytokines [57,61], and MMP/TIMP imbalance [62]. In DD+Mel, no morphologically detectable fibrosis was observed (0 points by light microscopy), demonstrating potent antifibrotic action via NF-κB inhibition, reduced TGF-β1 [57,63], and restored MMP/TIMP balance [64].

Morphometric analysis revealed that in DD, the nuclear area was reduced (very large effect), indicating suppressed transcriptional activity. In DD+Mel, the nuclear area at 24 months was larger than the control (very large positive effect), suggesting increased transcriptional activity (“active nuclei”) [65], which may explain preserved protein synthesis. The nuclear/cytoplasmic ratio (NCR) naturally decreased with age in the controls. In DD, the NCR at 24 months was extremely low (very large effect) due to hypertrophy under dystrophy. In DD+Mel, the NCR was significantly higher (large positive effect), indicating preserved function. Binucleated hepatocytes were extremely increased in DD (extreme effect sizes), reflecting persistent regenerative stress. In DD+Mel, binucleation returned to control levels by 24 months (medium effect), indicating that melatonin prevents chronic hepatocyte death.

Based on these data, we propose the following protective mechanisms of exogenous melatonin:

Antioxidant: Melatonin and its metabolites (AFMK, AMK) are potent endogenous antioxidants that act without regeneration—in both hydrophilic and lipophilic environments—neutralize ROS/RNS, and activate Nrf2/ARE [9,10,11,15,57,66,67]. Melatonin deficiency in DD promoted oxidative stress (elevated ALT, AST, LDH, and steatosis, fibrosis), while restoration in DD+Mel normalized these parameters.

Anti-inflammatory: Melatonin deficiency activates NF-κB and increases IL-1β, IL-6, and TNF-α [68], leading to HSC activation and fibrosis [13,57]. Melatonin inhibits the NLRP3 inflammasome [61]. The absence of fibrosis in DD+Mel (d = 4.50 vs. DD) confirms this effect.

Circadian restoration: Melatonin resynchronises peripheral clocks, normalizing clock gene expression (Bmal1, Clock, Per1-3, and Cry1-2) and their targets (SREBP-1c, FAS, and ACC), thereby correcting lipid and carbohydrate metabolism and protein synthesis [69,70].

Antifibrotic: Melatonin suppresses HSC activation via PDGF/TGF-β1 [71], reduces collagen I/III [72], and enhances collagen degradation via MMPs [68]. Complete absence of fibrosis in DD+Mel is direct evidence.

Mitochondrial support: Melatonin accumulates in mitochondria, reduces electron leakage, improves oxidative phosphorylation, stabilizes membrane potential, and prevents apoptosis [72,73,74]. Preserved nuclear area in DD+Mel suggests maintained mitochondrial function.

Our study confirms and extends previous work. Vinogradova and Anisimov (2013) showed that melatonin prevents metabolic syndrome in desynchronised rats, but their study was limited to 12 months and lacked histological analysis [23]. We demonstrate long-term (24 months) structural protection, including complete fibrosis prevention. Fonken et al. (2010) reported increased body weight and impaired glycemia under constant light, but they did not assess the liver or use melatonin [31]; we show that desynchronosis induces liver steatosis and fibrosis, which is prevented by melatonin. Kettner et al. (2016) identified disrupted SREBP-1c in NAFLD [20]; we show that exogenous melatonin restores these disorders long-term. Clinical studies and meta-analyses confirm that melatonin reduces ALT, AST, triglycerides, and cholesterol in NAFLD and metabolic syndrome [53,54]. Our study provides experimental support for these clinical observations and shows that long-term melatonin therapy can completely prevent liver fibrosis.

Effects on the central nervous system require separate analysis and are beyond the scope of this study.

Our results have practical implications for gerontology, hepatology, and preventive medicine, such as the following:

Chronic light pollution is a risk factor for accelerated metabolic aging and NAFLD, justifying hygienic recommendations to limit artificial light at night, especially for the elderly.

Melatonin replacement (10 mg/kg, five times/week) is an effective strategy for preventing metabolic disorders and liver fibrosis in chronic desynchronosis, normalizing biochemistry, preventing steatosis/fibrosis, and preserving relative liver weight and nuclear area (geroprotective effect).

The identified morphometric markers (reduced nuclear area, increased binucleated hepatocytes, and decreased NCR) may serve as early diagnostic criteria for desynchronosis-induced liver damage in preclinical and clinical studies.

**Practical conclusions.**

1. Chronic light pollution may be considered a risk factor. The obtained data suggest the potential need for hygienic recommendations, although direct translation to humans requires caution due to the extreme nature of the constant light model (760 lx).
2. Melatonin administration (10 mg/kg five times per week) can be considered an effective strategy for preventing metabolic disorders and liver fibrosis in chronic desynchronosis. Our data show that melatonin not only normalizes biochemical parameters but also completely prevents steatosis and fibrosis, as well as preserves relative liver weight and hepatocyte nuclear area (geroprotective effect).
3. The identified morphometric markers (decreased nuclear area, increased proportion of binucleated hepatocytes, and decreased NCR) can be used as early diagnostic criteria for desynchronosis-induced liver damage in preclinical and clinical studies.

## 5. Limitations of the Study

Despite the comprehensive approach and significance of the results obtained, this study has a number of limitations that should be considered when interpreting the data.

- **Use of only male animals.** This study was conducted on male rats to exclude the influence of the estrous cycle on biochemical parameters. However, this limits the extrapolation of the results to the female population since significant sex differences in melatonin metabolism and sensitivity to circadian disturbances are known [75].
- **Constant illumination model (760 lx/m^2^).** Although this is a classic model of desynchronosis, it represents an extreme variant of light pollution. Real human living conditions usually involve less pronounced but more complex circadian rhythm disturbances (shift work, evening use of gadgets, dim light at night, etc.). Further studies should use more physiological models (e.g., dim light at night, dLAN) [32,76].
- **Limited set of molecular markers.** In this study, we did not assess the following: (a) the expression of clock genes (Bmal1, Clock, Per1/2, and Cry1/2) in the liver, although previous studies have shown that constant illumination significantly changes the expression patterns of these proteins; (b) the activity of Nrf2 and its targets (SOD, GPx, catalase, and HO-1); (c) the level of pro-inflammatory cytokines (IL-1β, IL-6, and TNF-α) and NF-κB; (d) the activity of key lipogenesis enzymes (FAS, ACC, and SCD1); and (e) insulin, leptin, adiponectin levels and the HOMA-IR index. These data are necessary to finally confirm the proposed mechanisms.
- **Lack of data on food and water intake.** Individual food intake was not measured, so the contribution of nutritional factors to the observed metabolic effects cannot be completely ruled out. However, the decrease in body weight in the DD+Mel group (d = 0.35 when compared with the control at 24 months) with free access to food indicates a direct metabolic effect of melatonin rather than just a decrease in appetite.
- **Fixed dose of melatonin (10 mg/kg).** Other dosages were not studied, which does not allow us to determine the optimal therapeutic range and construct complete dose–response curves. However, the selected dose proved to be highly effective (d < 0.2 for most parameters).
- **Clinical translation.** As with any preclinical study, a direct translation of the results to humans requires caution. Melatonin metabolism in rats and humans differs, although the main pathways (hydroxylation in the liver) are similar. Doses effective in rats (10 mg/kg ≈ 2 mg/kg for humans according to the species rule) may not correspond to optimal doses for humans (usually 1–10 mg/day).

## 6. Conclusions and Findings

As a result of the conducted longitudinal 24-month study, it was established that:

- Chronic melatonin deficiency induced by constant illumination (light pollution) is a powerful independent risk factor for the development of systemic metabolic dysregulation, dysfunction, and structural changes in the liver. The identified complex of disorders includes hypertriglyceridemia, hypercholesterolemia, biphasic dysglycemia, hepatocyte cytolysis, cholestasis, hypoproteinemia, steatosis (d = 7.67), and pericellular fibrosis (d = 4.50).
- The phenomenon of metabolic disproportion in the DD group is described for the first time—a disproportionate increase in body weight compared to liver weight at a young age (d = 2.31)—reflecting a violation of the harmonious development of organs and systems.
- The geroprotective effect of melatonin involves not only the restoration of biochemical parameters to levels that do not differ statistically from the controls (d < 0.2 for most parameters), but also the structural protection of the liver: prevention of steatosis (d = 0.80 towards improvement), the absence of morphologically detectable fibrosis (0 points), preservation of relative liver weight (d = 1.60 positive), and an increase in hepatocyte nuclear area (d = 2.32 positive).
- The mechanisms of the protective action of melatonin include antioxidant defenses, anti-inflammatory effects, restoration of the circadian regulation of metabolism, antifibrotic effects, and maintenance of mitochondrial function.
- Chronic light-induced desynchronosis may serve as an experimental model of accelerated metabolic aging, which can be used for screening geroprotectors and studying the mechanisms of age-associated pathology.

## Figures and Tables

**Table 1:** Dynamics of melatonin levels in rat blood.

	6 Months	12 Months	18 Months	24 Months
**Control**	77.29 ± 8.59	57.85 ± 5.44	36.44 ± 3.21	11.91 ± 2.31
**DD**	15.18 ± 3.27 ***	8.97 ± 0.57 ***	7.25 ± 0.98 ***	5.15 ± 1.11 ***
**DD+Mel**	68.40 ± 5.2 *** ●●●	58.19 ± 8.46 *** ●●●	59.21 ± 10.25 *** ●●●	59.09 ± 9.21 *** ●●●

Note. *** (*p* ≤ 0.0005): in comparison with control values during the same period of ontogenesis; ●●● (*p* ≤ 0.0005): in comparison with DD group values during the same period of ontogenesis. In the control group, 30 rats were examined at 6 and 12 months, 22 rats at 18 months, and 18 rats at 24 months; in the DD group, 30 rats were examined at 6 and 12 months, 19 rats at 18 months, and 16 rats at 24 months; in the DD+MEL group, 30 rats were examined at 6, 12 and 18 months, and 20 rats at 24 months.

**Table 2:** Dynamics of body weight, liver weight, and relative liver weight with Cohen’s d (M ± SD).

Group		6 Months	12 Months	18 Months	24 Months
**Body weight (g)**	Control	359.8 ± 32.23	458.5 ± 54.41 ***	640.6 ± 59.85 ***	555.1 ± 69.56 **
	DD	503.5 ± 34.48 ▪▪▪	650.7 ± 33.98 *** ▪▪▪	710.4 ± 43.95 *** ▪▪▪	722.9 ± 73.56 ▪▪▪
	DD+Mel	400.3 ± 29.82 ▪▪▪ °°°	528.5 ± 48.69 *** ▪▪▪ °°°	595.4 ± 63.15 *** °°°	533.7 ± 50.71 °°°
	**Cohen’s d (DD vs. Control)**	4.30 (extreme)	3.99 (very large)	1.23 (very large)	2.34 (very large)
	**Cohen’s d (DD+Mel vs. Control)**	1.19 (large)	1.11 (very large)	0.75 (medium)	0.35 (small)
**Liver weight (g)**	Control	13.51 ± 1.23	16.35 ± 2.10 ***	22.28 ± 2.47 ***	18.12 ± 2.38 ***
	DD	16.65 ± 1.90 ▪▪▪	19.50 ± 3.57 ** ▪▪▪	24.96 ± 2.87 *** ▪▪▪	21.36 ± 2.72 *** ▪▪▪
	DD+Mel	14.01 ± 0.98 ▪▪▪ °°°	16.10 ± 2.32 *** °°°	27.76 ± 3.11 *** °°°	20.28 ± 1.95 ▪
	**Cohen’s d (DD vs. Control)**	1.98 (very large)	1.02 (large)	0.93 (large)	1.18 (very large)
	**Cohen’s d (DD+Mel vs. Control)**	0.45 (small)	0.11 (very small)	1.79 (very large) *	0.88 (large)
**Relative liver weight (arb. units)**	Control	3.76 ± 0.07	3.82 ± 0.06	3.66 ± 0.28 **	3.27 ± 0.029 ***
	DD	3.26 ± 0.49 ▪▪▪	2.99 ± 0.51 ▪▪▪	3.75 ± 0.55 ***	2.99 ± 0.56 ***
	DD+Mel	3.51 ± 0.29 ▪	3.06 ± 0.45 * ▪	3.82 ± 0.97 ***	3.83 ± 0.49 ▪▪▪ °°°
	**Cohen’s d (DD vs. Control)**	1.43 (very large)	2.31 (very large)	0.24 (small)	0.70 (medium)
	**Cohen’s d (DD+Mel vs. Control)**	0.89 (large)	2.10 (very large) *	0.22 (small)	1.60 (very large) *

**Notes:** * *p* < 0.05; ** *p* < 0.005; *** *p* < 0.0005 vs. previous period of the same group. ▪ *p* < 0.05; ▪▪▪ *p* < 0.0005 vs. Control. °°° *p* < 0.0005 vs. DD. Cohen’s d: <0.20—very small; 0.20–0.50—small; 0.50–0.80—medium; 0.80–1.20—large; 1.20–4.00—very large; ≥4.00—extreme. Asterisk (*) in DD+Mel vs. Control column indicates a positive effect (improvement vs. Control): for relative liver weight, an increase (geroprotective effect); for liver weight at 18 months, also an increase. In the control group, 30 rats were examined at 6 and 9 months, 22 rats at 18 months, and 18 rats at 24 months; in the DD group, 30 rats were examined at 6 and 9 months, 19 rats at 18 months, and 16 rats at 24 months; in the DD+MEL group, 30 rats were examined at 6, 9, and 18 months, and 20 rats at 24 months.

**Table 3:** Dynamics of serum biochemical parameters with Cohen’s d (M ± SD).

Parameter	Age	Group	M ± SD	Cohen’s d (DD vs. Control)	Cohen’s d (DD+Mel vs. Control)
**ALT (U/L)**	**6 months**	**Control**	42.24 ± 5.39	1.37 (very large)	0.09 (very small)
		**DD**	50.23 ± 6.28 ▪		
		**DD+Mel**	42.72 ± 4.38		
	**12 months**	**Control**	37.72 ± 4.89	2.36 (very large)	0.07 (very small)
		**DD**	49.09 ± 4.73 ▪▪		
		**DD+Mel**	37.41 ± 4.18		
	**18 months**	**Control**	46.62 ± 4.84	3.83 (very large)	0.60 (medium)
		**DD**	69.57 ± 6.98 *** ▪▪▪		
		**DD+Mel**	43.62 ± 5.29 ♦♦♦		
	**24 months**	**Control**	56.36 ± 10.88	2.60 (very large)	0.68 (medium)
		**DD**	83.39 ± 9.93 *** ▪▪▪		
		**DD+Mel**	50.38 ± 5.88 ♦♦♦		
**AST (U/L)**	**6 months**	**Control**	79.64 ± 9.68	1.00 (large)	0.60 (medium)
		**DD**	90.38 ± 11.67 ▪		
		**DD+Mel**	74.40 ± 5.26 ♦♦♦		
	**12 months**	**Control**	64.58 ± 6.59	2.27 (very large)	0.26 (small)
		**DD**	89.80 ± 14.29 *** ▪▪▪		
		**DD+Mel**	61.81 ± 12.06 ♦♦♦		
	**18 months**	**Control**	90.65 ± 11.52	2.02 (very large)	0.11 (very small)
		**DD**	122.8 ± 19.33 *** ▪▪▪		
		**DD+Mel**	91.92 ± 6.93		
	**24 months**	**Control**	111.60 ± 13.53	2.46 (very large)	0.97 (large)
		**DD**	151.30 ± 18.34 *** ▪▪▪		
		**DD+Mel**	98.00 ± 12.28 * ♦♦♦		
**LDH (U/L)**	**6 months**	**Control**	683.1 ± 53.7	3.56 (very large)	0.63 (medium)
		**DD**	947.7 ± 93.2 ▪▪▪		
		**DD+Mel**	750.0 ± 53.2 ♦♦♦		
	**12 months**	**Control**	778.5 ± 54.4	5.50 (extreme)	0.61 (medium)
		**DD**	1232.0 ± 101.8 *** ▪▪▪		
		**DD+Mel**	853.2 ± 77.4 ♦♦♦		
	**18 months**	**Control**	1111.0 ± 80.2	2.19 (very large)	0.59 (medium)
		**DD**	1374.0 ± 152.0 *** ▪▪▪		
		**DD+Mel**	1204.0 ± 101.7		
	**24 months**	**Control**	1493.0 ± 196.9	1.28 (very large)	0.15 (very small)
		**DD**	1703.0 ± 126.7 ***		
		**DD+Mel**	1458.0 ± 138.1 ♦♦♦		
**Total protein (g/L)**	**6 months**	**Control**	64.09 ± 5.14	3.46 (very large)	0.09 (very small)
		**DD**	47.27 ± 4.57 ▪▪▪		
		**DD+Mel**	63.56 ± 7.28 ♦♦♦		
	**12 months**	**Control**	68.32 ± 8.34	3.18 (very large)	0.17 (very small)
		**DD**	45.80 ± 5.54 ▪▪▪		
		**DD+Mel**	69.78 ± 8.39 ♦♦♦		
	**18 months**	**Control**	66.05 ± 6.05	5.33 (extreme)	0.38 (small)
		**DD**	36.81 ± 4.87 ▪▪▪		
		**DD+Mel**	68.30 ± 6.27 ♦♦♦		
	**24 months**	**Control**	61.42 ± 8.48	3.57 (very large)	0.04 (very small)
		**DD**	35.51 ± 5.78 ▪▪▪		
		**DD+Mel**	61.78 ± 8.0 ♦♦♦		
**Albumin (g/L)**	**6 months**	**Control**	49.03 ± 5.30	3.34 (very large)	0.20 (small)
		**DD**	33.24 ± 4.06 ▪▪▪		
		**DD+Mel**	48.04 ± 3.61 ♦♦♦		
	**12 months**	**Control**	39.27 ± 3.65	1.60 (very large)	0.14 (very small)
		**DD**	31.61 ± 6.0 * ▪▪		
		**DD+Mel**	40.08 ± 5.64 ♦♦♦		
	**18 months**	**Control**	33.11 ± 5.39	1.77 (very large)	0.62 (medium)
		**DD**	24.01 ± 5.05 * ▪▪▪		
		**DD+Mel**	36.69 ± 6.14		
	**24 months**	**Control**	28.79 ± 6.21	1.49 (very large)	0.48 (small)
		**DD**	19.18 ± 6.70		
		**DD+Mel**	31.89 ± 4.30 ♦♦♦		
**Total bilirubin (μ monthsl/L)**	**6 months**	**Control**	3.18 ± 0.29	2.06 (very large)	0.20 (small)
		**DD**	3.71 ± 0.24 ▪▪		
		**DD+Mel**	3.12 ± 0.30		
	**12 months**	**Control**	4.81 ± 0.27	0.13 (very small)	0.60 (medium)
		**DD**	4.86 ± 0.45		
		**DD+Mel**	4.68 ± 0.43		
	**18 months**	**Control**	5.65 ± 0.50	2.39 (very large)	0.30 (small)
		**DD**	6.89 ± 0.55 *** ▪▪		
		**DD+Mel**	5.48 ± 0.54		
	**24 months**	**Control**	6.69 ± 0.90	2.97 (very large)	0.23 (small)
		**DD**	9.16 ± 0.88 *** ▪▪▪		
		**DD+Mel**	6.88 ± 0.63		
**Direct bilirubin (μ monthsl/L)**	**6 months**	**Control**	1.00 ± 0.12	3.60 (very large)	0.18 (very small)
		**DD**	1.50 ± 0.15 ▪▪▪		
		**DD+Mel**	0.98 ± 0.09 ♦♦♦		
	**12 months**	**Control**	1.09 ± 0.11	4.22 (extreme)	0.60 (medium)
		**DD**	1.80 ± 0.23 *** ▪▪▪		
		**DD+Mel**	0.97 ± 0.10 ♦♦♦		
	**18 months**	**Control**	1.54 ± 0.16	4.37 (extreme)	0.36 (small)
		**DD**	2.40 ± 0.24 *** ▪▪▪		
		**DD+Mel**	1.41 ± 0.17		
	**24 months**	**Control**	2.45 ± 0.34	3.22 (very large)	0.21 (small)
		**DD**	3.55 ± 0.27 *** ▪▪▪		
		**DD+Mel**	2.52 ± 0.24		
**Glucose (m monthsl/L)**	**6 months**	**Control**	6.39 ± 0.49	2.43 (very large)	0.91 (large)
		**DD**	4.64 ± 0.89 ▪▪▪		
		**DD+Mel**	5.99 ± 0.87		
	**12 months**	**Control**	7.54 ± 1.15	1.22 (very large)	0.19 (very small)
		**DD**	9.23 ± 1.58 * ▪		
		**DD+Mel**	7.22 ± 0.66		
	**18 months**	**Control**	8.76 ± 1.41	1.21 (very large)	0.08 (very small)
		**DD**	10.85 ± 2.00 * ▪▪		
		**DD+Mel**	8.86 ± 1.24		
	**24 months**	**Control**	8.94 ± 2.44	1.67 (very large)	0.18 (very small)
		**DD**	12.56 ± 1.86 *		
		**DD+Mel**	8.61 ± 0.81		
**Triglycerides (m monthsl/L)**	**6 months**	**Control**	0.86 ± 0.044	6.40 (extreme)	0.00 (very small)
		**DD**	1.50 ± 0.14 ▪▪▪		
		**DD+Mel**	0.86 ± 0.12 ♦♦♦		
	**12 months**	**Control**	1.11 ± 0.10	6.12 (extreme)	0.10 (very small)
		**DD**	2.15 ± 0.22 *** ▪▪▪		
		**DD+Mel**	0.97 ± 0.09 ♦♦♦		
	**18 months**	**Control**	1.77 ± 0.17	4.25 (extreme)	0.19 (very small)
		**DD**	2.96 ± 0.35 *** ▪▪▪		
		**DD+Mel**	1.16 ± 0.13 *** ♦♦♦		
	**24 months**	**Control**	2.47 ± 0.34	1.78 (very large)	2.42 (very large)*
		**DD**	3.27 ± 0.54 *		
		**DD+Mel**	1.67 ± 0.32 * ♦♦♦		
**Cholesterol (mg/dL)**	**6 months**	**Control**	58.59 ± 4.83	3.97 (very large)	0.25 (small)
		**DD**	91.54 ± 10.70 ▪▪▪		
		**DD+Mel**	60.60 ± 5.09 ♦♦♦		
	**12 months**	**Control**	69.07 ± 7.87	4.18 (extreme)	0.13 (very small)
		**DD**	120.0 ± 15.32 *** ▪▪▪		
		**DD+Mel**	70.60 ± 7.16 ♦♦♦		
	**18 months**	**Control**	78.96 ± 8.25	4.59 (extreme)	0.09 (very small)
		**DD**	161.4 ± 23.98 *** ▪▪▪		
		**DD+Mel**	80.11 ± 7.58 ♦♦♦		
	**24 months**	**Control**	87.0 ± 13.83	4.28 (extreme)	0.09 (very small)
		**DD**	171.6 ± 24.29		
		**DD+Mel**	85.97 ± 9.42 ♦♦♦		

Notes: * *p* < 0.05; *** *p* < 0.0005 vs. previous period. ▪ *p* < 0.05; ▪▪ *p* < 0.005; ▪▪▪ *p* < 0.0005 vs. Control. ♦♦♦ *p* < 0.0005 vs. DD. Cohen’s d: <0.20—very small; 0.20–0.50—small; 0.50–0.80—medium; 0.80–1.20—large; 1.20–4.00—very large; ≥4.00—extreme. *—positive effect. In the control group, 30 rats were examined at 6 and 9 months, 22 rats at 18 months, and 18 rats at 24 months; in the DD group, 30 rats were examined at 6 and 9 months, 19 rats at 18 months, and 16 rats at 24 months; in the DD+MEL group, 30 rats were examined at 6, 9, and 18 months, and 20 rats at 24 months.

**Table 4:** Morphometric parameters of the liver with Cohen’s d (M ± SD).

Parameter	Age	Group	M ± SD	Cohen’s d (DD vs. Control)	Cohen’s d (DD+Mel vs. Control)
**Nuclear area (µm^2^)**	**6 months.**	**Control**	45.3 ± 3.1	2.56 (very large)	0.45 (small)
		**DD**	38.2 ± 2.9 ▪▪▪		
		**DD+Mel**	46.8 ± 3.4 °°°		
	**12 months.**	**Control**	48.7 ± 3.5	2.28 (very large)	0.40 (small)
		**DD**	40.5 ± 3.2 * ▪▪		
		**DD+Mel**	50.2 ± 3.8 ** °°°		
	**18 months.**	**Control**	52.1 ± 4.2	2.56 (very large)	0.51 (medium)
		**DD**	41.8 ± 3.6 ▪▪▪		
		**DD+Mel**	54.3 ± 4.1 *** °°°		
	**24 months.**	**Control**	48.6 ± 3.8	1.68 (very large)	2.32 (very large) *
		**DD**	42.3 ± 3.5 ▪▪▪		
		**DD+Mel**	58.4 ± 4.1 *** ▪▪▪ °°°		
**Nuclear-cytoplasmic ratio**	**6 months.**	**Control**	0.28 ± 0.02	1.67 (very large)	0.50 (medium)
		**DD**	0.32 ± 0.03 * ▪		
		**DD+Mel**	0.27 ± 0.02 °°		
	**12 months.**	**Control**	0.26 ± 0.02	0.67 (medium)	0.50 (medium)
		**DD**	0.28 ± 0.03 **		
		**DD+Mel**	0.25 ± 0.02 °		
	**18 months.**	**Control**	0.22 ± 0.02	0.00 (very small)	0.26 (small)
		**DD**	0.22 ± 0.03 ***		
		**DD+Mel**	0.23 ± 0.02 ***		
	**24 months.**	**Control**	0.18 ± 0.02	1.89 (very large)	1.15 (large) *
		**DD**	0.14 ± 0.02 *** ▪▪▪		
		**DD+Mel**	0.21 ± 0.02 *** ▪▪▪ °°°		
**Binucleated hepatocytes (%)**	**6 months.**	**Control**	5.3 ± 0.9	4.74 (extreme)	1.64 (very large)
		**DD**	11.2 ± 1.8 ▪▪▪		
		**DD+Mel**	7.8 ± 1.2 ** ▪▪ °		
	**12 months.**	**Control**	7.2 ± 1.1	4.83 (extreme)	1.38 (very large)
		**DD**	14.5 ± 2.1 *** ▪▪▪		
		**DD+Mel**	9.1 ± 1.4 *** ▪▪ °°°		
	**18 months.**	**Control**	6.1 ± 1.0	5.38 (extreme)	0.90 (large)
		**DD**	12.3 ± 1.9 *** ▪▪▪		
		**DD+Mel**	7.5 ± 1.1 °°°		
	**24 months.**	**Control**	4.8 ± 0.8	3.53 (very large)	0.72 (medium)
		**DD**	8.8 ± 1.5 *** ▪▪▪		
		**DD+Mel**	5.6 ± 0.9 °°°		
**Steatosis degree (points)**	**6 months.**	**Control**	0	3.90 (very large)	0.35 (small)
		**DD**	1.0 ± 0.3 ▪▪▪		
		**DD+Mel**	0.1 ± 0.1 °°°		
	**12 months.**	**Control**	0.3 ± 0.2	6.58 (extreme)	0.27 (small)
		**DD**	2.5 ± 0.4 *** ▪▪▪		
		**DD+Mel**	0.2 ± 0.1 °°°		
	**18 months.**	**Control**	0.5 ± 0.2	7.67 (extreme)	0.00 (very small)
		**DD**	2.8 ± 0.3 *** ▪▪▪		
		**DD+Mel**	0.5 ± 0.2 ** °°°		
	**24 months.**	**Control**	1.0 ± 0.2	7.20 (extreme)	0.80 (large) *
		**DD**	2.8 ± 0.3 *** ▪▪▪		
		**DD+Mel**	0.8 ± 0.3 ** °°°		
**Fibrosis severity (points)**	**6 months.**	**Control**	0	0	0
		**DD**	0		
		**DD+Mel**	0		
	**12 months.**	**Control**	0	2.50 (very large)	0
		**DD**	0.5 ± 0.2 ▪▪▪		
		**DD+Mel**	0 °°°		
	**18 months.**	**Control**	0	3.33 (very large)	0
		**DD**	1.0 ± 0.3 *** ▪▪▪		
		**DD+Mel**	0 °°°		
	**24 months.**	**Control**	0	4.50 (extreme)	0
		**DD**	1.8 ± 0.4 *** ▪▪▪		
		**DD+Mel**	0 °°°		

Notes: * *p* < 0.05; ** *p* < 0.005; *** *p* < 0.0005 vs. previous period.▪ *p* < 0.05; ▪▪ *p* < 0.005; ▪▪▪ *p* < 0.0005 vs. Control. ° *p* < 0.05; °° *p* < 0.005; °°° *p* < 0.0005 vs. DD. Cohen’s d: <0.20—very small; 0.20–0.50—small; 0.50–0.80—medium; 0.80–1.20—large; 1.20–4.00—very large; ≥4.00—extreme. * —positive effect (improvement compared to Control). In the control group, 30 rats were examined at 6 and 9 months, 22 rats at 18 months, and 18 rats at 24 months; in the DD group, 30 rats were examined at 6 and 9 months, 19 rats at 18 months, and 16 rats at 24 months; in the DD+MEL group, 30 rats were examined at 6, 9, and 18 months, and 20 rats at 24 months.

## Data Availability

The original contributions presented in this study are included in the article. Further inquiries can be directed to the corresponding author.
